# The Effect of Caffeine Consumption and Acute Withdrawal on Resting‐State fMRI Brain Connectivity, Mood and Cognition

**DOI:** 10.1111/ejn.70511

**Published:** 2026-04-19

**Authors:** Tatum Sevenoaks, Fiona Lancelotte, Nicholas Souter, Lorenzo Stafford, Charlotte Rae, Martin Yeomans

**Affiliations:** ^1^ School of Psychology University of Sussex Sussex UK; ^2^ Department of Psychology University of Portsmouth Portsmouth UK

**Keywords:** caffeine, fMRI, resting‐state, reward, withdrawal

## Abstract

Caffeine is the most widely consumed psychoactive substance, yet few studies have investigated how habitual and acute consumption and withdrawal impacts resting‐state brain connectivity. Notably, prior research lacks adequate control for deprivation state, despite evidence that caffeine reinforcement occurs primarily by alleviating withdrawal. This study used a between‐participant design to assess resting‐state fMRI brain connectivity, mood and cognition in three groups: (1) moderate consumers (200–500 mg/day) tested after overnight abstinence (caffeine withdrawn, CW); (2) moderate consumers tested after overnight abstinence followed by 100 mg of caffeine (caffeine not withdrawn [CNW]); and (3) non‐consumers of caffeine (< 50 mg/day, NC). Sixty healthy volunteers, aged 18–45 (*n* = 20/group) completed the Bond–Lader mood battery, a rapid visual information processing task and a resting‐state fMRI scan. For resting‐state brain connectivity, the CW group showed altered nucleus accumbens connectivity with primary visual cortex compared to CNW and NC groups. The CNW group showed stronger anterior insula connectivity with precuneus cortex compared to CW and NC groups. For network‐level analyses, the CNW group exhibited reduced limbic within‐network connectivity and altered connectivity between limbic and occipital cortex compared to CW and NC groups. The anterior salience network showed group differences in connectivity with the putamen, pallidum and thalamus. The supplementary somatomotor network showed greater connectivity with the bilateral putamen in both caffeine groups, but reduced connectivity with the right middle temporal gyrus for the CW group. No significant main group effect emerged for mood and cognition. These findings demonstrate that caffeine consumption and withdrawal produce distinct alterations in resting‐state brain connectivity.

## Graphical abstract

Habitual and acute caffeine consumption and acute caffeine withdrawal produce distinct alterations in resting‐state fMRI brain connectivity, in regions primarily associated with reward, interoception, emotion regulation, motor function and visual processing. These findings contribute towards the understanding of the caffeinated brain and the caffeine withdrawal state.

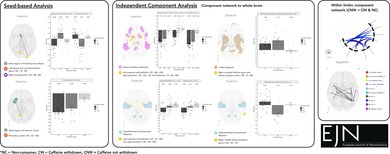

**Abbreviations**
ANOVA: analysis of variance
ANTs: advanced normalisation tools
AP: anterior‐to‐posterior
ART: artefact detection tools
BOLD: blood‐oxygen‐level‐dependent
CBF: cerebral blood flow
CNW: caffeine not withdrawn
CSF: cerebrospinal fluid
CW: caffeine withdrawn
DAN: dorsal attention network
DMN: default mode network
FDR: false discovery rate
fMRI: functional magnetic resonance imaging
GLM: general linear model
ICA: independent component analysis
LH: left hemisphere
MNI: Montreal Neurological Institute
MPRAGE: magnetization prepared gradient‐echo
NC: non‐consumer
PA: posterior‐to‐anterior
PET: positron emission tomography
RH: right hemisphere
ROI: region of interest
RVIP: rapid visual information processing
SPM: statistical parametric mapping

## 1 Introduction

Caffeine is the most widely consumed psychoactive substance globally (Nehlig et al. 1992), and a principal constituent of coffee and many types of tea, both of which are amongst the top choices of drinks people choose to consume apart from water (Reddy et al. 2024). Additionally, caffeine is added to many other foods and beverages, including many popular soft drinks and, more recently, energy drinks (Verster and Koenig 2018). Caffeine is well known and consumed for its stimulant properties, and generally, habitual consumers report enhanced alertness, mental performance, mood and energy (McLellan et al. 2016). Caffeine's primary mechanism of action is through non‐selective antagonism of adenosine A_1_ and A_2_ receptors in the brain (Fredholm et al. 1999). Adenosine is a neuromodulator that inhibits arousal and promotes sleep when bound predominantly to A_1_ receptors in the basal forebrain, limbic system, hypothalamus, brainstem and subarachnoid space (Porkka‐Heiskanen et al. 1997). By blocking adenosine binding to receptors, caffeine increases wakefulness and arousal (Ribeiro and Sebastião 2010). Habitual caffeine consumption has also been found to alter the adenosine system, increasing plasma adenosine concentration and upregulating adenosine receptors, resulting in tolerance towards the effects of caffeine (McLellan et al. 2016; Varani et al. 2000).

Although not typically considered a drug of abuse, regular caffeine use leads to dependence (Schuh and Griffiths 1997; Strain et al. 1994). Acute caffeine withdrawal in those dependent on caffeine can result in withdrawal symptoms, most commonly headache, fatigue and poor concentration; this is evident with just 100 mg/day—equivalent to roughly one cup of coffee (Schuh and Griffiths 1997). To date, research has demonstrated the characteristics of caffeine in terms of its withdrawal effects on mood, cognition and even preference for novel flavours (Chambers et al. 2007; Rogers et al. 2003; Tinley et al. 2003; Yeomans, Ripley, et al. 2002). Although heavily debated in the literature, much evidence suggests that the effects of caffeine are largely due to the alleviation of withdrawal symptoms, commonly referred to as the withdrawal reversal hypothesis (James and Rogers 2005). For instance, caffeine has been shown to increase cognitive performance in working memory tasks and mental alertness in habitual consumers given caffeine when deprived, but these effects were no longer found when consumers were given caffeine not deprived (Yeomans, Ripley, et al. 2002). Additionally, no net benefit of caffeine was found when given to habitual non‐consumers of caffeine, supporting the withdrawal reversal hypothesis (Rogers et al. 2003). Caffeine has also been shown to be a powerful reinforcer of liking for certain novel flavours associated with caffeine (Yeomans et al. 1998; Yeomans, Jackson, Lee, Nesic, and Durlach 2000). However, liking for novel flavoured drinks paired with caffeine is only acquired in a caffeine deprived state (Chambers et al. 2007; Tinley et al. 2003; Yeomans, Jackson, Lee, Steer, et al. 2000; Yeomans, Pryke, and Durlach 2002).

Whilst several studies have investigated habitual caffeine consumption and acute withdrawal on mood and cognition, fewer studies have investigated caffeine's impact on brain connectivity. This is a key knowledge gap to fill, given that the adenosine mechanism of caffeine underpins its mood and cognitive effects, as well as the effect of withdrawal, via the brain. Thus far, most studies have investigated caffeine's effects on brain function using task‐based fMRI. Studies employing working memory tasks have reported increased activation in the prefrontal cortex, the right anterior cingulate cortex and the striatum and reduced activation in the thalamus and hippocampus in habitual caffeine consumers following caffeine administration (Haller et al. 2014; Klaassen et al. 2013; Koppelstaetter et al. 2008; Lin et al. 2023). Notably, a study contrasting acute caffeine consumption and withdrawal found higher activity in the right middle frontal gyrus in the caffeine condition following caffeine consumption (Lin et al. 2023). However, Koppelstaetter et al. (2008) found no differences in brain activation between habitual consumers given caffeine and those acutely withdrawn. Additionally, increased blood‐oxygenation‐level‐dependent (BOLD) activation in habitual caffeine consumers given caffeine has been found in the left cerebellum, putamen, insula, thalamus and right primary motor cortex, alongside decreased BOLD deactivation in medial and lateral posterior cortical areas during a visuomotor task inducing attention (Park et al. 2014). High consumers of caffeine also showed increased BOLD signal change in the visual cortex compared to low consumers of caffeine during passive sensory stimulation (Laurienti et al. 2002).

To a lesser extent, habitual caffeine consumption and acute caffeine administration have also been shown to alter resting‐state brain connectivity (Magalhães et al. 2021; Picó‐Pérez et al. 2023; Rack‐Gomer and Liu 2012; Wong et al. 2012; Wu et al. 2014). Reduced inter‐hemispheric BOLD connectivity in the motor cortex was found after acute administration of caffeine (Rack‐Gomer et al. 2009; Rack‐Gomer and Liu 2012). Interestingly, Wu et al. (2014) also found decreased activation in the motor and visual cortex following caffeine administration in non‐habitual caffeine consumers who had not consumed caffeine for a 6‐month period. Caffeine also significantly enhances the anti‐correlations between the default mode network (DMN) and the task positive network at rest (Wong et al. 2012). More recently, Magalhães et al. (2021) found decreased connectivity in a network of cortical and subcortical regions, as well as the somatosensory and limbic networks, for caffeine consumers compared to non‐consumers of caffeine. Furthermore, decreased connectivity of the posterior DMN and increased connectivity of the visual and right executive control networks were found following acute caffeine administration in habitual consumers (Picó‐Pérez et al. 2023). Notably, however, none of these studies adequately controlled for acute caffeine withdrawal or included a sufficiently powered sample to robustly detect differences between non‐consumers of caffeine and habitual caffeine consumers in both acutely withdrawn and not‐withdrawn states.

This study aimed to address this gap by investigating habitual caffeine consumption and acute withdrawal on resting‐state fMRI brain connectivity, mood and cognition in the following three groups: (a) habitual moderate caffeine consumers (200–500 mg/day of caffeine) tested after overnight caffeine abstinence to establish acute withdrawal (caffeine withdrawn [CW] group); (b) habitual moderate caffeine consumers (200–500 mg/day of caffeine) tested after overnight caffeine abstinence followed by consumption of 100 mg of caffeine to establish acute caffeine consumption (caffeine not withdrawn [CNW] group); and (c) non consumers of caffeine, defined as never consuming coffee and whose total daily caffeine intake is less than 50 mg/day (non‐consumer [NC] group).

Based on previous evidence we hypothesised that (1) changes in mood, cognition and brain connectivity would be observed in habitual caffeine consumers (CW and CNW groups) versus non‐consumers of caffeine (NC) indicative of a long‐term chronic adaptation as a result of habitual caffeine consumption rather than an acute effect; (2) that acute administration of 100 mg of caffeine to habitual moderate caffeine consumers would alter mood, cognition and brain connectivity, with differences observed between the CNW group and the CW and NC groups, as well as an effect of acute withdrawal with differences observed between the CW and the CNW and NC groups; and (3) that both habitual caffeine use, acute administration and acute withdrawal will alter mood, cognition and brain connectivity, with differences observed between all three groups. Specifically for mood and cognition, we predicted that the CNW group would have a significantly faster reaction time and number of correct responses in the rapid visual information processing (RVIP) task as well as increased alertness compared to the CW and NC groups after breakfast. In contrast, we predicted that both caffeine consumer groups would have a significantly slower reaction time and number of correct responses as well as reduced alertness compared to the NC group before breakfast. For brain connectivity we sought to assess group differences in regions of interest (ROIs) most likely involved in caffeine reward and withdrawal (nucleus accumbens, hypothalamus and anterior insula) (Magalhães et al. 2021; Nehlig et al. 2010; Picó‐Pérez et al. 2023). Additionally, we aimed to explore whole‐brain connectivity differences between groups, with a specific interest in networks theoretically and previously implicated in habitual caffeine consumption and withdrawal: the anterior salience, executive control and somatomotor and limbic networks (Magalhães et al. 2021; Picó‐Pérez et al. 2023; Rack‐Gomer et al. 2009; Wu et al. 2014). Overall, this gives a more complete picture on the neural changes associated with long‐term and acute caffeine use while also crucially controlling for acute caffeine withdrawal.

## 2 Methods

### 2.1 Participants and Study Design

Sixty healthy volunteers aged 18–45 (*n* = 41 female and *n* = 19 male) were recruited, 20 into each of three groups: CNW, CW and NC. The sample size included in this study was based on effect sizes reported in previous studies using similar fMRI paradigms and is consistent with normative sample sizes in the field. Notably, the most recent study of resting state differences between coffee consumers and non‐consumers reported effects with *n* = 54 (Magalhães et al. 2021).

The study included minor deception and was advertised to potential participants as exploring the effects of breakfast on mood, cognition and brain connectivity to avoid any bias related to the knowledge of caffeine. All other details of the study procedure were described to participants accurately in advance of participation. Eligibility criteria included those aged 18–45 (there is an upper age limit as this study includes a measure of cognition that is age sensitive), not on any prescription medication other than oral contraceptives, who regularly (at least 4 days/week) consume breakfast, who do not smoke more than five cigarettes per week or have an aversion to any of the products served for breakfast in this study: wheat, dairy products or fruit conserves. Potential participants were screened for habitual caffeine consumption (see Table S1) and eligibility criteria via an online survey prior to being invited to participate (approved by the Sciences and Technology Cross‐Schools Research and Ethics Committee: ER/MARTIN/24). Participants recruited into groups CW and CNW habitually consumed at least one cup of coffee per day and had an estimated total daily caffeine consumption of 200–500 mg/day. Those consuming 100 mg of caffeine a day have been shown to be dependent on caffeine, and we therefore concluded that those moderately dependent on caffeine would consume between 200 and 500 mg/day; this is in alignment with previous studies (Laurienti et al. 2002). Those recruited into the NC group were non‐coffee drinkers and had an estimated total daily caffeine consumption of < 50 mg/day. Notably, we chose to recruit moderate caffeine consumers for the CNW and CW groups to maximise differences observed between the NC groups. This study used a between‐participant experimental design to contrast differences in brain connectivity, mood and cognition between the three groups: CNW, CW and NC. Double blinding of participants and the researcher was implemented for habitual moderate caffeine consumers, who were randomised into either the CW or CNW group. This study protocol was approved by the Brighton and Sussex Medical School Research and Ethics Committee (ER/MARTIN/22) and conformed to the British Psychological Society guidelines for ethical human research. All participants provided informed consent prior to beginning the study.

### 2.2 Procedure

Participants were tested in the morning between the hours of 08:00–10:30, and the duration of the testing session lasted no longer than 2 h. Upon arrival, participants completed the Bond–Lader mood task and the RVIP task. They then consumed a standard breakfast of two slices of toast (Hovis wholemeal bread), spread (sunflower spread or Lakeland butter) and a choice of two Ratton Pantry jams (apricot, strawberry, raspberry or blackberry) as well as a hot drink. To manipulate acute caffeine withdrawal state, participants were asked to abstain from eating and to drink only water from 22:00 pm on the evening before testing. For CNW participants, the hot beverage provided to them with their breakfast was a cup of caffeinated coffee. CW participants were provided with a cup of decaffeinated coffee as their hot beverage. Both caffeinated (Buenos Aires lungo—104 mg of caffeine) and decaffeinated coffee (Volluto decaffeinato—residual caffeine of 2 mg) was prepared using Nespresso original coffee pods dispensed into a Nespresso coffee machine, and participants had the option to add milk and sugar to their drink. The amount of milk and sugar added to the hot beverage was recorded by weighing the jug containing milk and the sugar bowl before and after breakfast. The amount of milk and sugar added was measured to assess any differences between caffeine consumer groups as some research suggests that there may be synergistic effects of sugar, in particular, with caffeine (Bernard et al. 2018; Reis et al. 2018). The NC group was given the choice of an herbal tea containing no caffeine to have with their breakfast. Participants were then transferred to the MRI centre, where they repeated the mood and cognitive tasks in a quiet room. After completing the mood and cognitive tasks, participants completed the resting‐state fMRI scan, which lasted 25 min (this was at least 45 min following caffeine or placebo consumption, to account for optimal caffeine absorption [Fredholm et al. 1999]). During the scan, participants were instructed to stay awake and maintain fixation on a cross back‐projected onto a screen. After the scanning session, participants were debriefed on the purpose of the study, where they were asked whether they believed the drink they consumed contained caffeine. Finally, participants were rewarded with either University of Sussex course credits or cash for their participation.

### 2.3 Study Measures

#### 2.3.1 Bond–Lader Mood Battery

Mood was assessed using an adapted computerised version of the validated Bond–Lader Visual Analogue Scales (Bond and Lader 1974), programmed using Qualtrics software. The mood battery consists of 16 scales representing different moods with opposite extremes. Participants were requested to position their bar on the rating line at the point that most accurately represents their current mood. Individual mood scales were scored from 0 to 100 and were subsequently combined into three mood factors: alertness, calmness and contentedness.

#### 2.3.2 RVIP Task

Cognitive performance was measured using the validated RVIP task (Bakan 1959), programmed using Inquisit software. Sustained attention and working memory are measured through the sequential presentation of numbers centrally on a screen at a rate of 80 numbers per minute. Participants were required to accurately detect and respond as quickly as possible to the target of three consecutive odd or even numbers, with eight target sequences occurring in each minute. The task included two 10‐minute tests divided into two 5‐minute blocks, separated by a brief rest. The number of correct responses and the mean reaction time for correct responses were recorded automatically.

#### 2.3.3 Resting‐State fMRI

##### 2.3.3.1 Resting‐State fMRI Parameters

Imaging was performed using a Siemens Prisma 3‐T MRI scanner. The scan session included a T1‐weighted magnetization prepared gradient‐echo (MPRAGE) structural scan (TE = 1.8/3.6/5.4/7.2 ms; 0.8 mm resolution, voxel size = 2 mm^3^), lasting 8 min 22 s and a resting‐state fMRI scan consisting of two spin echo field maps (2 mm resolution, anterior–posterior (AP) and posterior–anterior (PA)) lasting 18 s, as well as BOLD fMRI (2 runs: AP and PA; TR = 800 ms, TE = 37 ms, voxel size = 2 mm^3^) lasting 7 min 53 s per run, 15 min 46 s total. The two scan sequences were taken from the Human Connectome Project Development and Aging protocol (Harms et al. 2018). The total duration of the scanning session was approximately 25 minutes.

##### 2.3.3.2 Resting‐State fMRI Preprocessing and Denoising

Resting‐state fMRI data were analysed using CONN (Whitfield‐Gabrieli and Nieto‐Castanon 2012) (RRID:SCR_009550) release 22.v2407 (Nieto‐Castanon and Whitfield‐Gabrieli 2022), and SPM (RRID:SCR_007037) release 12.7487 (Friston et al. 2006) in MATLAB.

First, field maps were applied to unwarp functional data using custom in‐house MATLAB scripts. The unwarped files were imported into CONN, with each run (AP, PA phase encoding) of the resting‐state functional data set‐up as two sessions. Functional and anatomical data were preprocessed using a modular preprocessing pipeline (Nieto‐Castanon 2020) including realignment, outlier detection, direct segmentation and MNI‐space normalisation and smoothing. Functional data were co‐registered to a reference image (first scan of the first session) using a least squares approach and a six‐parameter (rigid‐body) transformation (Friston et al. 2006) and resampled using b‐spline interpolation. Potential outlier scans were identified using ART (Whitefield‐Gabrieli et al. 2011) as acquisitions with framewise displacement above 0.9 mm or global BOLD signal changes above 5 standard deviations (Nieto‐Castanon 2022; Power et al. 2014), and a reference BOLD image was computed for each subject by averaging all scans excluding outliers. Functional and anatomical data were normalised into standard MNI space, segmented into grey matter, white matter and cerebrospinal fluid (CSF) tissue classes and resampled to 2 mm isotropic voxels following a direct normalisation procedure (Calhoun et al. 2017; Nieto‐Castanon 2022) using SPM unified segmentation and normalisation algorithm (Ashburner 2007; Ashburner and Friston 2005) with the default IXI‐549 tissue probability map template. Last, functional data were smoothed using spatial convolution with a Gaussian kernel of 5 mm full width at half maximum.^1^

In addition, functional data were denoised using a standard denoising pipeline (Nieto‐Castanon 2020) including the regression of potential confounding effects characterised by white matter time series (5 CompCor noise components), CSF time series (5 CompCor noise components), motion parameters and their first‐order derivatives (12 factors) (Friston et al. 1996), outlier scans (below 59 factors) (Power et al. 2014), session (run) effects and their first‐order derivatives (2 factors) and linear trends (2 factors) within each functional run, followed by bandpass frequency filtering of the BOLD time series (Hallquist et al. 2013) between 0.008 and 0.09 Hz. CompCor (Behzadi et al. 2007; Chai et al. 2012) noise components within white matter and CSF were estimated by computing the average BOLD signal as well as the largest principal components orthogonal to the BOLD average, motion parameters and outlier scans within each subject's eroded segmentation masks. From the number of noise terms included in this denoising strategy, the effective degrees of freedom of the BOLD signal after denoising were estimated to range from 107.2 to 145.1 (average 143.3) across all subjects (Nieto‐Castanon 2022).

### 2.4 Data Analysis

This section details pre‐registered data analysis as described in the OSF project (osf.io/tqe7f). Initial analysis compared key demographic data (age and biological sex) between the three groups as well as habitual caffeine consumption between groups CW and CNW.

#### 2.4.1 Mood and Cognition

Prior to statistical analysis, RVIP reaction time data were checked, and reaction times of less than 250 ms were recorded as false hits considering that this reaction time would be highly unlikely. The remaining reaction times were then averaged for each participant at each test time (before and after breakfast). Data were checked for outliers using visual inspection of boxplots, as well as calculating z‐scores, with a cut off score of ±3. Differences between groups were investigated by fitting a general linear model (GLM) and contrasted using ANOVA. GLM assumptions of normality, homoscedasticity and linearity were assessed; in cases where the model did not meet assumptions, a robust GLM was fitted. First, we investigated differences between non‐consumers and caffeine consumers for all measures at the pre‐breakfast baseline. We then assessed group differences for the post‐breakfast score for all measures controlling for baseline scores. RVIP data for one subject in the NC group was missing, and therefore, this participant was excluded from the RVIP analysis. Data analyses were conducted in R using RStudio Version 2024.12.1 + 653. GLMs were conducted in base R and interpreted using the Broom and Parameters packages (Lüdecke et al. 2020; Robinson et al. 2025); the standard *p* < 0.05 criteria were used for determining significance.

#### 2.4.2 Resting‐State fMRI

##### 2.4.2.1 Seed‐Based Connectivity Analysis

For first‐level analysis, seed‐based connectivity maps and ROI‐to‐ROI connectivity matrices were estimated, characterising the patterns of functional connectivity with five ROIs: the hypothalamus (taken from the atlas by Neudorfer et al. 2020), left and right nucleus accumbens (taken from the Harvard–Oxford subcortical structural atlas^2^) and left and right anterior insula (taken from the atlas by Faillenot et al. 2017). All ROI masks were first binarised, and then the masks for the hypothalamus and anterior insula were transformed into 2 mm space using advanced normalisation tools (ANTs) (Tustison et al. 2021). Functional connectivity strength was represented by Fisher‐transformed bivariate correlation coefficients from a weighted‐GLM (Nieto‐Castanon 2020), defined separately for each possible pair of the three ROIs, modelling the association between their BOLD signal time series. To compensate for possible transient magnetisation effects at the beginning of each run, individual scans were weighted by a step function convolved with an SPM canonical haemodynamic response function and rectified.

Group‐level analyses were performed using a GLM (Nieto‐Castanon 2020). For each individual voxel, a separate GLM was estimated, with first‐level connectivity measures at this voxel as dependent variables (one independent sample per subject) and the caffeine groups as independent variables. Voxel‐level hypotheses were evaluated using multivariate parametric statistics with random effects across subjects and sample covariance estimation across multiple measurements. Inferences were performed at the level of individual clusters (groups of contiguous voxels). Cluster‐level inferences were based on parametric statistics from Gaussian random field theory (Nieto‐Castanon 2020; Worsley et al. 1996). Results were thresholded using a cluster‐forming *p* < 0.001 voxel‐level threshold and then a familywise‐corrected p‐FDR < 0.05 cluster‐size threshold (Chumbley et al. 2010).

##### 2.4.2.2 Independent Component Analysis

Group‐level independent component analyses (group‐ICA) (Calhoun et al. 2001) were performed to estimate 20 temporally coherent networks from the fMRI data combined across all subjects. For first‐level analysis, the BOLD signal from every timepoint and voxel in the brain was concatenated across subjects and resting‐state runs along the temporal dimension. A singular value decomposition of the z‐score normalised BOLD signal (subject‐level SVD) with 64 components separately for each subject was used as a subject‐specific dimensionality reduction step. The dimensionality of the concatenated data was further reduced using a singular value decomposition with 20 components and a fast‐ICA fixed‐point algorithm (Hyvarinen 1999) with hyperbolic tangent (G1) contrast function to identify spatially independent group‐level networks from the resulting components. Finally, GICA3 back‐projection (Erhardt et al. 2011) was used to compute ICA maps associated with these same networks separately for each individual subject.

Within‐network connectivity differences between groups were investigated by creating and exporting an ICA parcellation ROI file for each component that best represented the networks of interest (pre‐registered): limbic, executive control, anterior salience and somatomotor (primary and supplementary), as well as remaining primary resting‐state networks: default mode, dorsal attention and visual networks (not pre‐registered). Components were spatially matched according to the Yeo 7 Networks and Yeo 17 Networks (Thomas Yeo et al. 2011), and exported from CONN. Each component file was then split into separate files of clusters over 10 voxels that constitute different regions of the network based on the Schaefer parcellation (7‐network 100 parcels) (Schaefer et al. 2018). Files of the different regions of each network were imported back into CONN as separate ROIs; connectivity matrices were then estimated to investigate within‐network connectivity using each possible node‐to‐node connection within a given network, and differences were investigated between groups. In addition, group differences for component networks to whole brain were investigated. Group‐level analyses were once again performed using a GLM (Nieto‐Castanon 2020), with the same approach as described for seed‐based connectivity analysis.

### 2.5 Exploratory Analysis

Additional exploratory analysis (not pre‐registered) also investigated group differences for other key variables measured within this study. Specifically, the amount of milk and sugar added to the hot drink provided to participants was assessed between caffeine consumer groups (CW and CNW) as some research suggests that there may be synergistic effects of sugar with coffee. Additionally, we assessed differences between caffeine consumer groups regarding whether they believed that the drink they consumed contained caffeine. This was to assess whether there were any group differences in caffeine associated expectation effects. Lastly, for the ICA, we also assessed within‐network and network to whole‐brain connectivity for the remaining main resting state networks: visual network, dorsal attention network (DAN) and the DMN.

## 3 Results

### 3.1 Participant Summary

Participant characteristics are summarised in Table 1. All three groups did not differ significantly in terms of age (*F*_2,57_ = 2.376, *p* = 0.102) and caffeine consumption (mg/day) did not differ significantly between CNW and CW (*F*_1,36_ = 1.584, *p* = 0.216). As expected, habitual caffeine consumption did differ significantly between caffeine consumers (CNW and CW) and the NC group (*F*_1,58_ = 340.9, *p* < 0.001). Sex distribution was not significantly different between groups (X^2^ = 1.08, *p* = 0.583).

**TABLE 1:** Participant characteristics.

	NC		CNW		CW	
	Mean	SD	Mean	SD	Mean	SD
Age (years)	19.6	1.72	20.45	1.72	21.05	2.67
Caffeine consumption (mg/day)	6.75	14.07	309.25	66.65	282	70.23

Abbreviations: CNW = caffeine not withdrawn, CW = caffeine withdrawn, NC = no caffeine.

### 3.2 Mood and Cognition

No significant differences between caffeine consumers (CW and CNW groups combined) and non‐consumers (NC) were observed for baseline performance for any measure (Table 2). In addition, there were no significant differences for the main effect of group on any of the mood and cognitive measures post‐breakfast whilst controlling for baseline scores. However, post hoc tests contrasting the CNW and NC groups revealed a significant difference in mean reaction time (*T*(50) = 2.273 [−68.533, −4.230], *p* = 0.027) (Figure S1) and approached significance when contrasting the CW and CNW groups (*T*(50) = 1.970 [−0.639, 66.535], *p* = 0.054). Notably, the number of incorrect responses at baseline (*F*_2,551_ = 0.384, *p* = 0.683) and post‐breakfast (*F*_2,50_ = 1.545, *p* = 0.223) did not differ significantly between groups. There were five participants who were considered outliers based on the number of false hits and mean reaction time scores and were therefore not included in the RVIP analysis. Considering none of the mood and cognitive measures showed significant differences for the main effect of group, we did not include these measures as covariates in the subsequent resting‐state fMRI analyses.

**TABLE 2:** Baseline scores for all mood and cognitive measures between caffeine consumers and non‐consumers.

	Non‐consumers		Caffeine consumers		ANOVA
	Mean	SD	Mean	SD	*p*
Alertness	51.99	15.91	52.56	14.87	0.891
Contentedness	66.62	11.55	63.24	12.50	0.315
Calm	61.75	19.64	56.61	14.70	0.26
Mean reaction time	576.71	58.10	596.19	97.04	0.752
Number of correct responses	42.58	12.32	37.53	15.8	0.294

### 3.3 Resting‐State fMRI Seed‐Based Analysis

**FIGURE 1:**
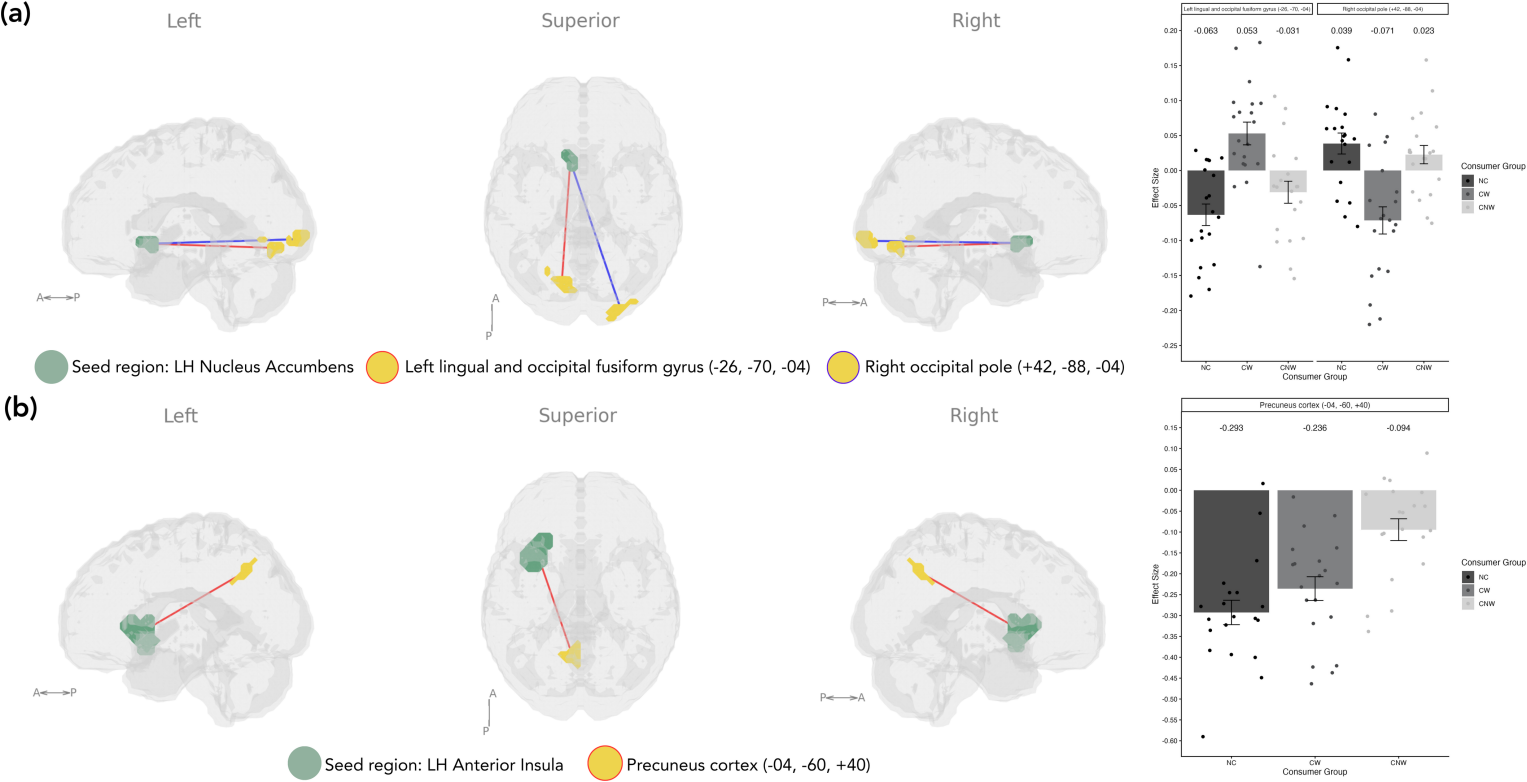
Seed‐voxel analysis indicating significant group differences for (a) LH nucleus accumbens with reduced connectivity between the right occipital pole and increased connectivity between the left lingual and occipital fusiform gyrus contrasting the CW group with both NC and CNW groups and (b) LH anterior insula with increased connectivity between the precuneus cortex contrasting the CNW group with both CW and NC groups. CNW = caffeine not withdrawn, CW = caffeine withdrawn, LH = left hemisphere, NC = no caffeine.

For seed‐voxel analysis (Table 3), an overall significant difference was found between groups for left hemisphere nucleus accumbens connectivity with the right occipital pole (*F*_2,57_ = 14.53, *p* < 0.001) and left lingual and occipital fusiform gyrus (*F*_2,57_ = 12.27, *p* = 0.019). Specifically, the CW group had lower connectivity between the left nucleus accumbens and the right occipital pole compared to the NC group (*T*(57) = 5.07, *p* < 0.001) and NC and CNW groups combined (*T*(57) = 5.19, *p* < 0.001) and higher connectivity with the left lingual gyrus and occipital fusiform gyrus compared to the NC group (*T*(57) = 5.10, *p* < 0.001) and NC and CNW groups combined (*T*(57) = 5.18, *p* < 0.001) (Figure 1a). In addition, the CNW group had significantly higher connectivity between the left anterior insula and the precuneus cortex compared to the NC group (*T*(57) = 5.28, *p* = 0.001), as well as the NC and CW groups combined (*T*(57) = 4.96, *p* = 0.011) (Figure 1b), although the group‐level *F*‐test was not significant. There were no significant group differences in seed‐voxel hypothalamus connectivity. Additionally, there were no significant group differences from the ROI‐to‐ROI analysis (hypothalamus, nucleus accumbens and anterior insula).

**TABLE 3:** Significant group differences for seed‐voxel connectivity analysis.

Seed	Contrast	Cluster regions	MNI coordinates	Number of voxels	p‐FDR
Nucleus accumbens (LH)	*F*‐test (group effects)	Right occipital pole (79%), lateral occipital cortex (21%)	+42, −88, −04	133	< 0.001
		Left lingual gyrus (75%), left occipital fusiform gyrus (19%), left cerebellum 6 (2%), not labelled (4%)	−10, −76, −14	57	0.019
	CW < NC	Right occipital pole (81%), lateral occipital cortex (19%)	+38, −90, −06	190	< 0.001
	CW < NC and CNW	Right occipital pole (77%), lateral occipital cortex (23%)	+42, −88, −04	241	< 0.001
	CW > NC	Left lingual gyrus (60%), left occipital fusiform gyrus (26%), left cerebellum 6 (3%), not labelled (10%)	−10, −76, −14	144	< 0.001
	CW > NC and CNW	Left lingual gyrus (52%), left occipital fusiform gyrus (27%), left cerebellum 6 (5%), not labelled (17%)	−26, −70, −04	126	< 0.001
Anterior insula (LH)	CNW > NC	Precuneus cortex (83%), cingulate gyrus (7%), not labelled (11%)	−04, −60, +40	150	0.001
	CNW > NC and CW	Precuneus cortex (78%), cingulate gyrus (22%)	−04, −60, +40	108	0.011

*Note:* Each cluster is made up of a percentage of different regions within that cluster as reflected by the percentage values included.

Abbreviations: CNW = caffeine not withdrawn, CW = caffeine withdrawn, LH = left hemisphere, NC = no caffeine.

### 3.4 Independent Component Analysis

For the independent component analysis (ICA), five components most representative of the networks of interest were identified: limbic, executive control, anterior salience and somatomotor (primary and supplementary). We investigated group differences for both within‐network connectivity and network to whole‐brain connectivity.

**FIGURE 2:**
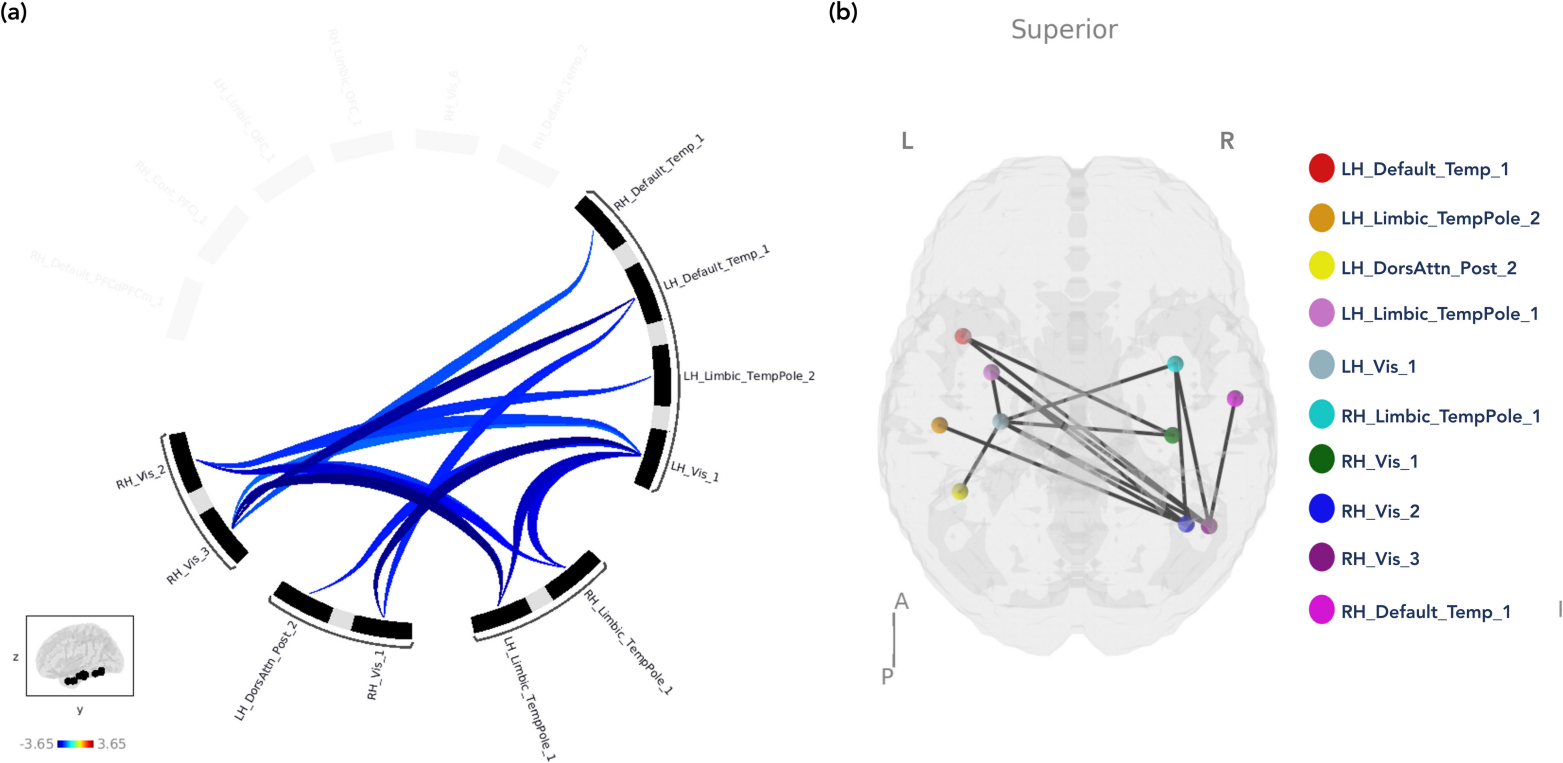
The ICA component that best represented the limbic network that differed significantly when contrasting CNW vs CW and NC displayed as (a) a connectome ring and (b) nodes of the network on a MNI template brain presented from the superior view. DorsAttn = dorsal attention, LH = left hemisphere, Post = posterior, RH = right hemisphere, Temp = temporal, TempPole = temporal pole, Vis = visual.

Within‐network connectivity analysis revealed group differences for the ICA component that best represented the limbic network only (Figure 2). Specifically, the CNW group had one significant cluster comprising of three node‐to‐node connections with lower within‐network connectivity compared to the NC group (*F*_2,56_ = 6.33, *p* = 0.049). The CW group also had two significant clusters of node‐to‐node connections with higher within‐network connectivity compared to the CNW group (*F*_2,56_ = 6.31, *p* = 0.045; *F*_2,56_ = 5.60, *p* = 0.045). Additionally, the CNW group had four significant clusters of node‐to‐node connections with lower within‐network connectivity than both the CW and NC groups combined (*F*_2,56_ = 8.41, *p* = 0.009; *F*_2,56_, *p* = 0.013; *F*_2,56_ = 5.61, *p* = 0.029; *F*_2,56_ = 4.77, *p* = 0.046) (Table 4). No significant within‐network group differences were found for any of the other networks of interest.

**TABLE 4:** Significant within‐network group differences for the ICA component best representative of the limbic network showing clusters of node‐to‐node connections for each significant group contrast.

Contrast	Clusters of node‐to‐node connections	p‐FDR
CNW < NC	RH_Vis_1–LH_Vis_1 RH_Vis_1–LH_Default_Temp_1 LH_DorsAttn_Post_2–LH_Vis_1	0.049
CW > CNW	RH_Vis_1–LH_Vis_1 LH_DorsAttn_Post_2–LH_Default_Temp_1	0.045
	RH_Vis_3–LH_Limbic_TempPole_1 RH_Vis_2–LH_Limbic_TempPole_1 RH_Vis_2–RH_Limbic_TempPole_1 RH_Vis_3–RH_Limbic_TempPole_1	0.045
CNW < CW and NC	RH_Vis_1–LH_Vis_1 LH_DorsAttn_Post_2–LH_Vis_1 RH_Vis_1–LH_Default_Temp_1	0.009
	RH_Vis_3–LH_Limbic_TempPole_1 RH_Vis_2–LH_Limbic_TempPole_1 RH_Vis_2–RH_Limbic_TempPole_1 RH_Vis_3–RH_Limbic_TempPole_1	0.013
	RH_Vis_3–LH_Default_Temp_1 RH_Vis_2–LH_Limbic_TempPole_2 RH_Vis_2–LH_Vis_1 RH_Vis_3–RH_Default_Temp_1 RH_Vis_3–LH_Vis_1	0.029
	LH_Limbic_TempPole_1–LH_Vis_1 RH_Limbic_TempPole_1–LH_Vis_1	0.046

*Note:* Clusters of node‐to‐node connections depict the number of connections between different regions of the ICA component that best represents the limbic network that differs significantly between groups. Centroid coordinates of regions included in this table are available at https://github.com/ThomasYeoLab/CBIG/blob/master/stable_projects/brain_parcellation/Schaefer2018_LocalGlobal/Parcellations/MNI/Centroid_coordinates/Schaefer2018_100Parcels_7Networks_order_FSLMNI152_2mm.Centroid_RAS.csv.

Abbreviations: CNW = caffeine not withdrawn, CW = caffeine withdrawn, DorsAttn = dorsal attention, LH = left hemisphere, NC = no caffeine, Post = posterior, RH = right hemisphere, Temp = temporal, TempPole = temporal pole, Vis = visual.

**FIGURE 3:**
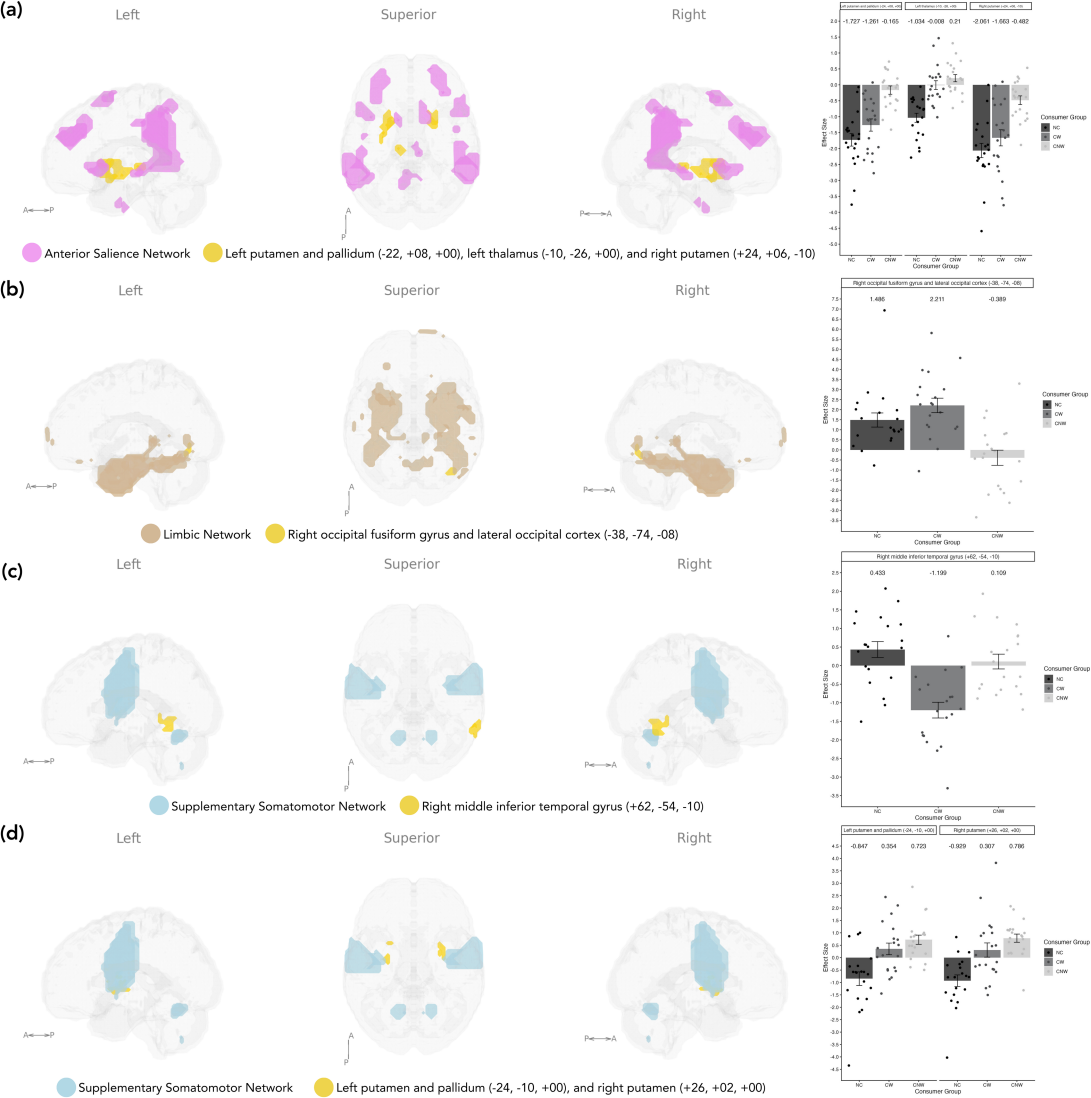
Significant clusters depicted in yellow associated with ICA components that best represent the networks of interest showing differences between groups contrasting (a) all groups (*F*‐test) for the anterior salience network, (b) all groups (*F*‐test) for the limbic network, (c) CW < CNW and NC for the supplementary somatomotor network and (d) CW and CNW > NC for the supplementary somatomotor network. CNW = caffeine not withdrawn, CW = caffeine withdrawn, NC = no caffeine.

In addition, when investigating component networks of interest to whole‐brain connectivity (Figure 3 and Table 5), connectivity between the anterior salience network and the left putamen and pallidum (*F*_2,57_ = 20.98, *p* < 0.001), right putamen (*F*_2,57_ = 15.34, *p* < 0.001) and left thalamus (*F*_2,57_ = 25.56, *p* = 0.04) differed overall between groups. Specifically, the CNW group had higher connectivity between the anterior salience network and the left putamen and pallidum compared to the NC group (*T*(57) = 6.20, *p* < 0.001), the CW group (*T*(57) = 5.08, *p* = 0.004) and both NC and CW groups combined (*T*(57) = 6.00, *p* < 0.001). Higher connectivity between the anterior salience network and the right putamen was also shown for the CNW group compared to the NC group (*T*(57) = 5.38, *p* < 0.001) and both NC and CW groups combined (*T*(57) = 5.43, *p* < 0.001). Additionally, connectivity between the anterior salience network and the left thalamus was higher for the CNW group compared to the NC group (*T*(57) = 7.07, *p* < 0.001). Lastly, both CW and CNW groups had significantly higher connectivity between the anterior salience network and the left putamen and pallidum (*T*(57) = 7.00, *p* = 0.004) and left thalamus (*T*(57) = 5.23, *p* = 0.037) compared to the NC group.

Connectivity between the limbic network and the right occipital fusiform gyrus differed overall between groups (*F*_2,57_ = 13.66, *p* = 0.037). The CW group had higher connectivity between the limbic network and the right occipital fusiform gyrus compared to the CNW group (*T*(57) = 5.19, *p* = 0.003). Additionally, both the CW and NC groups combined had higher connectivity between the limbic network and right occipital fusiform gyrus compared to the CNW group (*T*(57) = 5.00, *p* = 0.001).

Lastly, significant group differences were found for the supplementary somatomotor network; lower connectivity with the right middle temporal gyrus was found for the CW group compared to the NC group (*T*(57) = 5.39, *p* = 0.014) and NC and CNW groups combined (*T*(57) = 5.77, *p* = 0.008). Additionally, higher connectivity with the left putamen was shown for the CNW group compared to the NC group (*T*(57) = 5.10, *p* = 0.003), and higher connectivity with the left (*T*(57) = 4.84, *p* = 0.019) and right putamen (*T*(57) = 5.16, *p* = 0.019) was shown for both CW and NCW groups compared to the NC group.

No significant differences were found between groups for the executive control and primary somatomotor networks and the rest of the brain.

**TABLE 5:** Significant between group connectivity differences for ICA components that best represent the networks of interest to whole brain.

Network	Contrast	Cluster regions	MNI coordinates	Number of voxels	p‐FDR
Anterior salience	*F*‐test	Left putamen (71%), left pallidum (8%), left amygdala (4%), frontal orbital cortex (1%), not labelled (16%)	−22, +08, +00	400	< 0.001
		Right putamen (58%), right amygdala (5%), not labelled (28%)	+24, +06, −10	186	< 0.001
		Thalamus (93%), not labelled (7%)	−10, −26, +00	55	0.043
	CNW > NC	Left putamen (63%), left pallidum (8%), left caudate (3%), left amygdala (3%), thalamus (2%), frontal orbital cortex (1%), not labelled (21%)	−22, +08, +00	642	< 0.001
		Right putamen (69%), right amygdala (5%), right frontal orbital cortex (1%), not labelled (25%)	+30, −06, −04	337	< 0.001
		Left thalamus (84%), brainstem (1%), not labelled (15%)	−10, −26, +00	74	0.019
	CNW > CW	Left putamen (70%), left pallidum (10%), left amygdala (5%), not labelled (15%)	−24, +08, +00	129	0.005
	CNW and CW > NC	Left putamen (92%), left pallidum (8%)	−22, +08, +00	131	0.004
		Left thalamus (91%), brainstem (1%), not labelled (8%)	−10, −26, +00	74	0.038
	CNW > NC and CW	Left putamen (60%), left pallidum (8%), left caudate (4%), left amygdala (3%), left thalamus (2%), not labelled (21%)	−22, +08, +00	607	< 0.001
		Right putamen (64%), right amygdala (5%), right frontal orbital cortex (1%), not labelled (29%)	+24, +06, −10	334	< 0.001
Limbic	*F*‐test	Right occipital fusiform gyrus (38%), lateral occipital cortex (35%), right cerebellum crus 1 (1%), not labelled (26%)	+38, −74, −08	80	0.037
	CW > CNW	Right occipital fusiform gyrus (47%), lateral occipital cortex (27%), right cerebellum crus 1 (2%), right cerebellum 6 (1%), not labelled (23%)	+38, −74, −08	152	0.003
	CW and NC > CNW	Right occipital fusiform gyrus (46%), lateral occipital cortex (30%), right cerebellum crus 1 (2%), right cerebellum 6 (1%), not labelled (21%)	+36, −76, −08	173	0.001
Supplementary somatomotor	CW < NC	Right middle temporal gyrus (63%), inferior temporal gyrus (28%), lateral occipital cortex (4%), not labelled (5%)	+62, −60, −06	99	0.014
	CW < NC and CNW	Right middle temporal gyrus (60%), inferior temporal gyrus (35%), lateral occipital cortex (1%), not labelled (4%)	+62, −54, −10	111	0.008
	CNW > NC	Left putamen (85%), left pallidum (11%), not labelled (3%)	−24, +00, +04	124	0.003
	CW and CNW > NC	Left putamen (82%), left pallidum (18%)	−24, −10, +00	79	0.019
		Right putamen (96%), not labelled (4%)	+26, +02, +00	77	0.019

Abbreviations: CNW = caffeine not withdrawn, CW = caffeine withdrawn, NC = no caffeine.

### 3.5 Exploratory Analysis

Exploratory analysis investigating other key study variables found no significant differences between caffeine consumer groups CNW and CW for milk (*F*_1,38_ = 0.007, *p* = 0.932) or sugar added (*F*_1,38_ = 0.185, *p* = 0.669). Furthermore, no differences between caffeine consumer groups were observed regarding whether they believed the drink they consumed contained caffeine (X^2^ < 0.001, *p* = 1).

**FIGURE 4:**
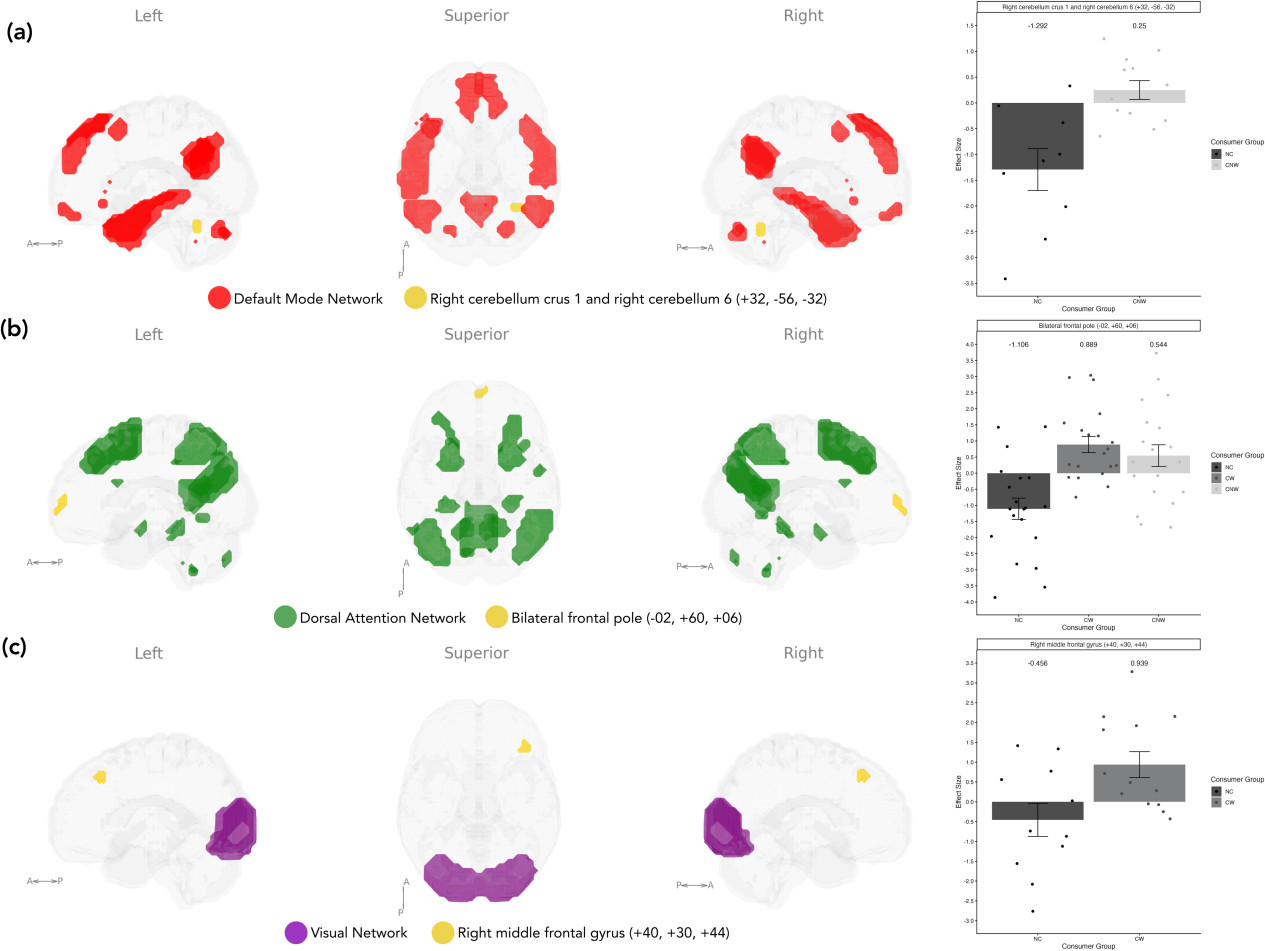
Significant clusters associated with ICA components that best represent remaining resting‐state networks showing differences between groups contrasting (a) CNW > NC for the default mode network (DMN), (b) CW and CNW > NC for the dorsal attention network (DAN) and (c) CW > NC for the visual network. CNW = caffeine not withdrawn, CW = caffeine withdrawn, DAN = dorsal attention network, DMN = default mode network, NC = no caffeine.

In addition, exploratory analysis investigated the remaining main resting‐state networks: visual network, DAN and the DMN (Figure 4 and Table 6). No within‐network group differences were observed for these networks. However, when investigating network to whole‐brain connectivity, the CNW group had significantly higher connectivity between the DMN and the right cerebellum compared to the NC group (*T*(57) = 5.56, *p* = 0.035). In addition, for the DAN, the CW group had significantly higher connectivity with the bilateral frontal pole compared to the NC group (*T*(57) = 4.64, *p* = 0.015), and both CW and CNW groups had significantly higher connectivity with the bilateral frontal pole and left paracingulate gyrus compared to the NC group (*T*(57) = 4.81, *p* = 0.006). Lastly, the CW group had significantly higher connectivity than the NC group between the primary visual network and the right middle frontal gyrus (*T*(57) = 4.92, *p* = 0.037).

**TABLE 6:** Exploratory ICA investigating significant between group connectivity differences from components that best represent the remaining resting‐state networks to whole brain.

Network	Contrast	Cluster region	MNI coordinates	Number of voxels	p‐FDR
DMN	CNW > NC	Right cerebellum crus 1 (66%), right cerebellum 6 (32%), not labelled (2%)	+32, −56, −32	82	0.035
DAN	CW > NC	Right frontal pole (58%), left frontal pole (20%), not labelled (22%)	+00, +60, +04	96	0.015
	CW and CNW > NC	Right frontal pole (46%), left frontal pole (31%), left paracingulate gyrus (1%), not labelled (22%)	−02, +60, +06	112	0.006
Visual	CW > NC	Right middle frontal gyrus (100%)	+40, +30, +44	79	0.037

Abbreviations: CNW = caffeine not withdrawn, CW = caffeine withdrawn, DAN = dorsal attention network, DMN = default mode network, NC = no caffeine.

## 4 Discussion

Caffeine has consistently been shown to impact mood and cognitive performance, with more recent evidence describing the effects of caffeine on brain connectivity (Magalhães et al. 2021; Picó‐Pérez et al. 2023; Rack‐Gomer and Liu 2012; Wong et al. 2012; Wu et al. 2014). Findings in this study build upon these previous studies by demonstrating for the first time robust characterisation of resting‐state functional connectivity alterations associated with acute caffeine withdrawal. We identified distinct connectivity patterns amongst habitual caffeine consumers, both acutely withdrawn and not withdrawn, as well as non‐consumers of caffeine.

Our seed‐based analysis revealed that caffeine consumers during acute withdrawal (CW) displayed altered connectivity from the nucleus accumbens to the primary visual cortex relative to both non‐consumers of caffeine (NC) and habitual caffeine consumers not withdrawn (CNW), demonstrating a neural mechanism unique to withdrawal. Prior research has implicated a similar network including the striatal nodes and thalamus, which notably have a very high density of A_2_A and A_1_ adenosine receptors, respectively, along with cerebellar and motor regions following caffeine administration (Magalhães et al. 2021; Svenningsson et al. 1999). Our finding suggests that during caffeine withdrawal, habitual consumers exhibit a shift in how the reward system interacts with the visual system. The reduced connectivity to higher‐order visual regions (left lingual and fusiform gyrus) in withdrawn caffeine consumers may reflect a blunted motivational influence of visual information, whereas increased connectivity to early visual cortex regions (right occipital pole) may indicate compensatory sensory responsiveness. This pattern of brain connectivity may correspond to withdrawal symptoms including reduced pleasure in engaging stimuli, modulated by a reduction in dopamine transmission, as well as heightened sensory sensitivity or distractibility, which is more commonly seen in caffeine withdrawal (Juliano and Griffiths 2004). Studies investigating caffeine's effects on the visual system have reported both enhanced activity in response to a visual stimulus in caffeine consumers given caffeine or placebo (Laurienti et al. 2002) and decreased connectivity in visual cortices in non‐habitual consumers given caffeine withdrawn (Wu et al. 2014). In contrast to our study, these findings demonstrate a long‐term and an acute effect of caffeine on the visual system, respectively. Our findings add to this existing literature demonstrating a nuanced effect in how caffeine withdrawal adapts visual processing.

In addition, we observed increased connectivity from the anterior insula to the precuneus cortex, a central hub of the DMN (Utevsky et al. 2014), in the CNW group compared to both the CW and NC groups. Considering the role of the anterior insula in interoception, salience detection and switching between the DMN and executive control network (Menon and Uddin 2010; Molnar‐Szakacs and Uddin 2022), this finding may support the hypothesis that caffeine plays a role in modulating DMN‐related activity, shifting the brain towards an externally focused or task‐related state (Childs and de Wit 2006; Picó‐Pérez et al. 2023). Notably, cognitive functions commonly altered with caffeine intake including episodic memory and visuospatial processing have been reported to involve the precuneus (Cavanna and Trimble 2006). Given that this difference does not occur in the CW group, the **i**ncreased coupling of these two regions is likely due to caffeine's acute effects rather than long‐term chronic changes as a result of habitual consumption. This aligns with prior research reporting greater activation in the precuneus in non‐consumers given caffeine in comparison to habitual consumers given caffeine (Gramling et al. 2018). However, this pattern of connectivity is inconsistent with much of the literature reporting a reduction in insula, DMN and precuneus activity following acute caffeine consumption (Haller et al. 2013, 2014; Kahathuduwa et al. 2018; Picó‐Pérez et al. 2023; Wu et al. 2014), as well as caffeine enhancing anti‐correlations between the DMN and the task positive network during eyes closed (Wong et al. 2012).

Considering the role of the hypothalamus in homeostasis, endocrine and autonomic nervous system regulation (Lechan and Toni 2000), we hypothesised to see group differences in this region. However, no significant group differences were found. This might simply be due to the very small size of the hypothalamus, and even though we combined both hemispheres into one seed region for this analysis, the voxel size and sequence parameters used in this study may not have been sensitive enough to pick up signal in this region. Notably, one study has shown activation in the bilateral hypothalamus in high daily coffee consumers (Nehlig et al. 2010). Additionally, there were no significant group differences for the ROI‐to‐ROI analysis. Although we hypothesised that there would likely be connectivity differences for these regions, we refrained from providing specific predictions given the variability in the literature as to how these regions might connect with each other in addition to the rest of the brain. Nevertheless, this finding suggests that instead of a combined mechanism integrating these three regions, parallel changes in connectivity between these regions and the rest of the brain better explain differences seen between groups. However, it may be possible that the relationship between these regions was not strong enough to be observed given our sample size.

ICA identified further connectivity alterations in networks previously implicated in habitual caffeine use. When investigating between‐group connectivity differences for (pre‐registered) networks of interest to whole brain, we found connectivity differences between the anterior salience network and regions associated with motor or reward relay systems, primarily the putamen and thalamus (Haber 2016; Marcuse et al. 2025). Greater connectivity was found for the CNW group compared to both CW and NC groups, consistent with previous research in turn supporting caffeine's role in enhancing alertness and reorienting attention towards novel stimuli, particularly motor and cognitive information (Park et al. 2014). However, some studies have reported decreased thalamic activation following caffeine administration, interpreted as increased arousal requiring less input from the thalamus (Klaassen et al. 2013; Portas et al. 1998). Notably, the elevated connectivity in the CW group relative to the NC group may reflect a residual upregulation of salience‐motor circuits that has not yet returned to baseline after acute withdrawal. Even the expectation of caffeine has been shown to induce dopaminergic responses in the thalamus measured using positron emission tomography (PET) (Kaasinen et al. 2004), supporting the observed increase in activity in these regions for those withdrawn compared to non‐consumers. However, no significant group differences were observed for anterior salience within‐network connectivity.

We also identified increased connectivity between the supplementary somatomotor network and the bilateral putamen for both CW and CNW groups, but with a greater extent for the CNW group. In addition to once again supporting caffeine's acute role in priming the motor system and increasing reward sensitivity (Ferré 2008), this finding also reflects long‐term changes due to chronic caffeine consumption. Conversely, the CW group showed decreased connectivity between the supplementary somatomotor network and the middle temporal gyrus, an area associated with integrating sensory input with stored knowledge and perception of motion (Xu et al. 2015). A disruption in this connectivity may explain withdrawal symptoms like difficulty concentrating or processing information as well as altered sensory perception (Juliano and Griffiths 2004). Although most prior studies report reduced motor and somatosensory connectivity following caffeine administration (Magalhães et al. 2021; Picó‐Pérez et al. 2023; Rack‐Gomer et al. 2009; Wu et al. 2014), these did not account for acute withdrawal, implying that effects are likely representative of chronic adaptation. For example, decreased somatomotor–prefrontal cortex connectivity reported after caffeine administration (Picó‐Pérez et al. 2023) may instead represent long‐term changes. A decrease in connectivity in the somatosensory network following caffeine consumption has been suggested to represent a more efficient pattern of connection with respect to motor control and alertness (Magalhães et al. 2021). However, in contrast to our findings, Wu et al. (2014) reported decreased motor cortex connectivity in caffeine consumers given caffeine compared to those withdrawn. Notably, we did not find significant group differences for the primary somatomotor network to whole brain. Additionally, we did not find significant within‐network connectivity group differences for either the primary or supplementary somatomotor networks.

As found by Magalhães et al. (2021), a reduction in limbic network connectivity was observed in the CNW group. Specifically, a decrease in connectivity to the right occipital fusiform gyrus was most evident between CNW and CW groups. This finding may imply that during withdrawal, systems involved in determining emotional responses re‐engage with visual sensory processing, potentially to process stimuli relevant to internal states like craving, as has been demonstrated with other addictive substances (Artiges et al. 2009; Sinha and Li 2007). In addition, a significant reduction in within‐network connectivity was also found for the CNW group, once again highlighting caffeine's acute impact on the limbic system. Notably, significant clusters within this network also included regions mostly within the visual network, reflecting the results from network to whole‐brain connectivity differences, but also regions in the DAN, DMN, and control networks. Given that the networks used were derived from sample‐specific ICA, it follows that they do not entirely align with respective networks as defined in other pre‐existing parcellations, although the analysis benefits from ensuring the network regions investigated derive most clearly from the participants' own regional fMRI signal.

Contrary to our hypothesis, no significant within‐network or network to whole‐brain group differences were found for the executive control network. Although there is limited evidence investigating caffeine's effect on the executive control network at rest, a study by Picó‐Pérez et al. (2023) found increased connectivity of the right dorsolateral prefrontal cortex within the right executive control network following coffee consumption. Additionally, studies have found increased activation of the frontoparietal network during working memory tasks (Haller et al. 2013; Koppelstaetter et al. 2008). Notably, a study investigating the effects of methylphenidate, modafinil and caffeine on resting‐state fMRI did, however, find that connectivity between the frontoparietal network and the DMN was modulated by the stimulant condition compared to placebo (Becker et al. 2022).

Exploratory ICA of other key resting‐state networks revealed additional group differences. Increased connectivity was found between the DMN and cerebellar regions in the CNW group compared to the NC group, a pattern inconsistent with previous findings of reduced DMN connectivity following caffeine administration (Picó‐Pérez et al. 2023; Wong et al. 2012; Wu et al. 2014). Additionally, increased connectivity between the DAN and the bilateral frontal pole regions in both the CW and CNW groups was observed compared to the NC group, which may be indicative of impaired executive control of attention in habitual caffeine consumers; however, further investigation is needed to validate this. Additionally, the NC group showed decreased connectivity between the primary visual network and the right middle frontal gyrus compared to the CW group but not compared to the CNW group. This aligns with our findings of increased connectivity between the nucleus accumbens and early visual cortex regions for the CW group described previously. No significant group differences were observed for within‐network connectivity for any of these networks.

No significant differences were observed between groups for any of the mood and cognitive measures in this study. Contrary to our hypotheses, we did not see any effect of caffeine withdrawal, which has been shown previously to impair attention (Lin et al. 2023; Yeomans, Ripley, et al. 2002). However, the CNW group had significantly faster reaction times in the RVIP task compared to the NC group, and the differences in scores approached significance when comparing the CNW to the CW group. This aligns with previous research indicating caffeine's acute effect in improving sustained attention and reaction time both in studies using the RVIP task (Haskell et al. 2005; Smit and Rogers 2000; Warburton 1995) as well as studies using other reaction time tasks (Lieberman et al. 1987; Smith et al. 1994, 1999). However, some studies have reported no effect of caffeine on reaction time (Loke et al. 1985), and even an impaired effect (Childs 1978). Contrary to our findings, most studies report altered mood, particularly alertness, following caffeine consumption in both a deprived and not deprived state (Stafford et al. 2006). However, research investigating the effect of caffeine on mood is complex, with several factors accounting for its effect (Hachenberger et al. 2025). Notably, unlike previous studies conducted in our lab, the RVIP task was programmed using Inquisit software, and reaction times were noticeably slower across all participants in this study compared to previous studies (Yeomans, Ripley, et al. 2002); therefore, the presentation of the task may have influenced the performance of the participants. In addition, it may not have been possible to detect caffeine effects for mood and cognitive outcomes considering that the sample size used in this study is small in comparison to other studies detecting effects for these measures. It is also important to note that any potential expectation effects as a result of receiving decaffeinated coffee may have further contributed towards no differences being observed between the CNW and CW groups.

Although an important strength of this study is the inclusion of both caffeine consumers withdrawn and not withdrawn compared to non‐consumers, several limitations should be noted. First, due to ethical implications, we could not include a group of non‐consumers who were administered caffeine, a manipulation that would have allowed us to isolate the effect of acute consumption in the absence of chronic consumption. Second, caffeine is known to reduce cerebral blood flow (CBF) and increase baseline cerebral metabolic rate of oxygen consumption (Griffeth and Buxton 2011), causing neurovascular uncoupling (Chen and Parrish 2009a; Perthen et al. 2008). However, neurovascular uncoupling effecting the BOLD response is not likely to occur with only 100 mg of caffeine (the amount provided in this study), as these effects are seen more robustly with higher doses of caffeine (Chen and Parrish 2009a, 2009b; Shepley et al. 2025). Furthermore, it is particularly unlikely to see such effects in moderate habitual caffeine consumers who have likely developed a physical tolerance to these effects (Kennedy and Haskell 2011). Nevertheless, future research would benefit from the simultaneous acquisition of CBF and BOLD signal. Third, by using coffee as a means to control for acute caffeine intake and withdrawal, we are unable to account for possible effects of other active coffee constituents as well as the placebo effect of expecting to receive caffeinated coffee (Flaten et al. 2003). Fourth, although we did obtain verbal confirmation from all participants that they had not eaten or drank anything other than water from 22:00 pm the night before the testing session to assess compliance with abstinence, the funding for this study did not afford the collection of biological samples to confirm caffeine exposure. Furthermore, to fully assess withdrawal symptoms, additional questions regarding other common withdrawal symptoms such as headache would have been beneficial to include within the Bond–Lader mood assessment. Fifth, interindividual differences in caffeine metabolism and absorption regarding known genetic variances in adenosine receptors may have impacted behavioural and neural responses (Nehlig 2018) and therefore should be taken into consideration for future research. Notably, the sample included in this study is a narrow young cohort, and therefore, caution should be taken when generalising to other age groups. Lastly, although the interpretations of these results have been carefully considered in the context of the literature, we acknowledge that such inferences may not be deductively valid, and future research should consider Bayesian analysis to validate these findings (Poldrack 2006).

The present findings demonstrate that both acute and chronic caffeine consumption and acute withdrawal impact resting‐state brain connectivity. Future research would benefit from a more comprehensive look at the effects of caffeine following extended withdrawal periods, thereby clarifying long‐term adaptations associated with habitual consumption and withdrawal. Additionally, future research using task‐based fMRI would be beneficial to fully understand any associations between behavioural responses and brain connectivity. Lastly, to further understand the nature of reward and withdrawal in habitual caffeine consumers, future work should investigate brain connectivity in response to caffeine‐related cues.

## 5 Conclusion

In conclusion, the data presented in this study has highlighted caffeine's multifaceted impact across various neural systems. Acute withdrawal was uniquely associated with altered reward to visual connectivity. Additionally, distinct patterns of connectivity were observed for acute caffeine consumption versus long‐term changes in regions primarily involved with interoception, emotion regulation and motor function. Importantly, the current findings provide additional evidence for alternative theories regarding the motivation to consume caffeine. One theory has emphasised withdrawal reversal as a form of negative reinforcement, proposing that caffeine consumption is primarily driven by alleviating withdrawal symptoms (James and Rogers 2005). In contrast, positive reinforcement models propose that caffeine produces a net beneficial effect and is consumed independent of withdrawal reversal (Addicott and Laurienti 2009). Considering that we have demonstrated a distinct pattern of connectivity for withdrawal as well as acute and long‐term caffeine consumption, it is likely that instead of a dichotomy of two opposing theories, the motivation to consume caffeine may involve neural mechanisms reflecting both negative and positive reinforcement. In summary, by controlling for acute caffeine withdrawal, we were able to draw stronger conclusions about not only the mechanisms underlying habitual and acute caffeine consumption but also the differences between the caffeinated brain and the caffeine withdrawal state.

## Author Contributions

**Tatum Sevenoaks:** conceptualization, project administration, methodology, data curation, formal analysis, writing – original draft, writing – review and editing. **Fiona Lancelotte:** data curation, writing – review and editing. **Nicholas Souter:** methodology, writing – review and editing. **Lorenzo Stafford:** writing – review and editing. **Charlotte Rae:** methodology, writing – review and editing. **Martin Yeomans:** conceptualization, supervision, writing – review and editing.

## Funding

This work was supported by the Biotechnology and Biological Sciences Research Council (BB/T008768/1).

## Ethics Statement

This study was conducted according to the guidelines of the Declaration of Helsinki, approved by the Brighton and Sussex Medical School research and ethics committee (ER/MARTIN/22) and conformed to the British Psychological Society guidelines for ethical human research. Informed consent was provided by all participants.

## Conflicts of Interest

The authors declare no conflicts of interest.

## Supporting information


**Figure S1:** The CNW group showed significantly faster mean reaction time at post breakfast compared to groups NC and CW.
**Table S1:** Caffeine consumption questionnaire.

## Data Availability

This project was pre‐registered on the OSF (osf.io/tqe7f) on 14 October 2022. Please note additions and changes to the preregistration in the Supporting Information. Raw mood and cognitive data can be accessed via the University of Sussex repository Figshare (https://figshare.com/s/da2438d4d1f4ce1c795f). Analyses scripts along with files for all regions of interest, component networks and associated significant clusters that were used to create all the figures included in this manuscript can be accessed on the OSF at https://doi.org/10.17605/OSF.IO/8JU76. Raw fMRI and pre‐processed data are available upon reasonable request from the corresponding author due to privacy and ethical restrictions.

## Endnotes

^1^
Sections of the above paragraph and paragraphs below were generated automatically by CONN toolbox with the intention that users should copy and paste this text verbatim into the methods section of the manuscript.

^2^
Available on Neurovault (https://identifiers.org/neurovault.collection:262).
