# Supplementary material for: The Effect of Caffeine Consumption and Acute Withdrawal on Resting‐State fMRI Brain Connectivity, Mood and Cognition
Source: Eur J Neurosci. 2026 Apr 19;63(8):e70511. doi: 10.1111/ejn.70511 (PMC13092888; doi:10.1111/ejn.70511)
Supplement: Supplementary file 1 — Figure S1: The CNW group showed significantly faster mean reaction time at post breakfast compared to groups NC and CW. Table S1: Caffeine consumption questionnaire. [file EJN-63-0-s001.docx]

**The effect of caffeine consumption and acute withdrawal on resting-state fMRI brain activity, mood, and cognition.**

**Supplementary Materials**

Tatum Sevenoaks^1^, Fiona Lancelotte^1^, Nicholas Souter^1^, Lorenzo Stafford^2^, Charlotte Rae^1^, Martin Yeomans^1^,

1 School of Psychology, University of Sussex

2 Department of Psychology, University of Portsmouth

Deviations from preregistration

This project was preregistered on the Open Science Framework (<https://doi.org/10.17605/OSF.IO/8JU76>) on (October 14^th^ 2022).

Deviations were made from this preregistration. (1) For fMRI resting-state seed-based analysis we aimed to focus on regions of interest including the nucleus accumbens, anterior insula, and hypothalamus. To note, we separated the masks for the nucleus accumbens and anterior insula by hemispheres but considering that the size of the hypothalamus is a considerably small region we decided to not separate this mask into left and right hemispheres. Therefore, in total this provided us with 5 regions of interest: left and right nucleus accumbens, left and right anterior insula, and the hypothalamus. (2) For fMRI independent component analysis, we aimed to look at the following networks: anterior salience, executive control, somatosensory and limbic. However, upon further consideration to improve thoroughness we decided it would be appropriate to also conduct exploratory analysis looking at the remaining main resting-state networks including the default mode, dorsal attention and the visual networks.


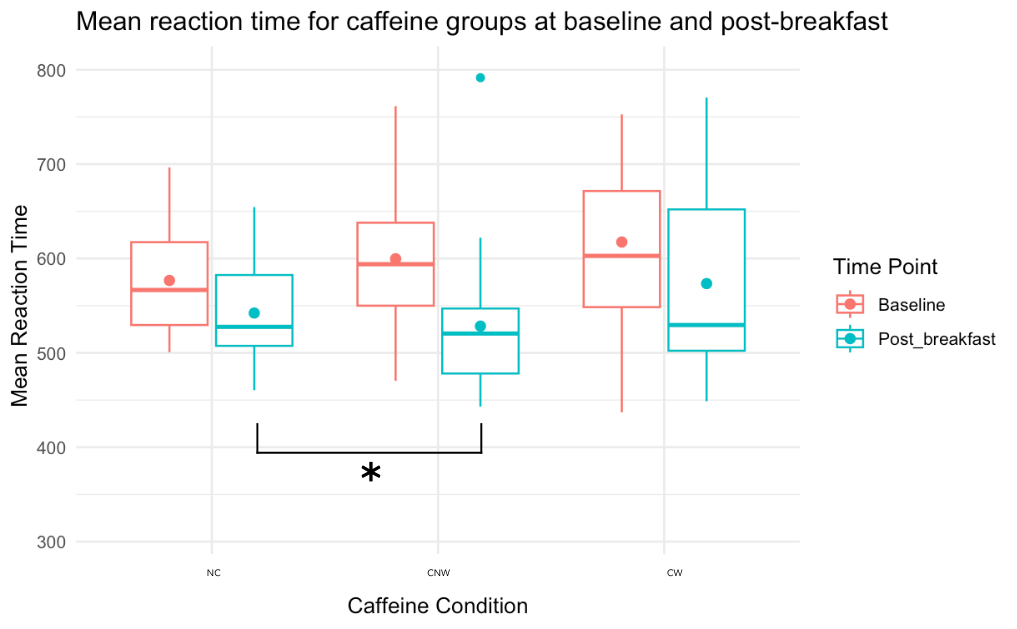


Abbreviations: NC = no caffeine, CW = caffeine withdrawn, CNW = caffeine not withdrawn.

Supplementary Figure 1: The CNW group showed significantly faster mean reaction time at post breakfast compared to groups NC and CW.

## *Supplementary Table 1: Caffeine consumption questionnaire.*

| **Question 1** | How many servings (e.g. cup, mug, glasses) of each drink do you consume each day? - Fruit juice |
| --- | --- |
| **Question 2** | How many servings (e.g. cup, mug, glasses) of each drink do you consume each day? - Filter coffee (e.g. espresso, latte, cappuccino etc.) |
| **Question 3** | How many servings (e.g. cup, mug, glasses) of each drink do you consume each day? - Instant coffee |
| **Question 4** | How many servings (e.g. cup, mug, glasses) of each drink do you consume each day? - Regular tea |
| **Question 5** | How many servings (e.g. cup, mug, glasses) of each drink do you consume each day? - Green tea |
| **Question 6** | How many servings (e.g. cup, mug, glasses) of each drink do you consume each day? - Fruit tea |
| **Question 7** | How many servings (e.g. cup, mug, glasses) of each drink do you consume each day? - Hot chocolate |
| **Question 8** | How many servings (e.g. cup, mug, glasses) of each drink do you consume each day? - Cola drinks |
| **Question 9** | How many servings (e.g. cup, mug, glasses) of each drink do you consume each day? - Other soft drinks |
| **Question 10** | How many servings (e.g. cup, mug, glasses) of each drink do you consume each day? - Milk |
| **Question 11** | How many servings (e.g. cup, mug, glasses) of each drink do you consume each day? - Energy drinks (please note below) |

The caffeine content (mg) for each drink used to calculate total caffeine consumption was as follows: fruit juice = 0, filter coffee = 120, instant coffee = 70, regular tea = 60, green tea = 30, fruit tea = 0, hot chocolate = 10, cola drinks = 35, other soft drinks = 0, milk = 0, energy drinks differed depending on what energy drink the participant specified. The caffeine content of each drink was then multiplied by the frequency at which the participant indicated that they consumed that drink ranging from: 0 (never) – 7 (more than 7 a day).
